# Editorial: The Clinical Application of Neoantigens

**DOI:** 10.3389/fimmu.2022.842633

**Published:** 2022-03-03

**Authors:** Jian-Guo Zhou, Zhenyu Ding, Huashan Shi, Min Cheng

**Affiliations:** ^1^ Department of Oncology, The Second Affiliated Hospital of Zunyi Medical University, Zunyi, China; ^2^ Translational Radiobiology, Department of Radiation Oncology, Universitätsklinikum Erlangen, Erlangen, Germany; ^3^ Department of Radiation Oncology, Universitätsklinikum Erlangen, Erlangen, Germany; ^4^ Comprehensive Cancer Center Erlangen-Europäische Metropolregion Nürnberg (EMN), Erlangen, Germany; ^5^ Department of Biotherapy, Cancer Center, West China Hospital, Sichuan University, Chengdu, China; ^6^ Department of Physiology, Weifang Medical University, Weifang, China

**Keywords:** neoantigens, editorial, clinical application, immune check inhibitor, predictive biomarker

To a large extent, the specificity of cancer immunotherapy is dependent on the recognition of specific tumor antigens, especially neoantigens. Neoantigens are newly formed antigens that have not been previously recognized by the immune system. Neoantigens can arise from altered tumor proteins formed as a result of tumor mutations or from viral proteins. They are highly restricted to tumor cells, with minimally established immune tolerance. Neoantigens used in vaccines and other types of immunotherapies are being studied in the treatment of many types of cancer. Furthermore, a growing body of evidence indicates that neoantigen-specific T cells underlie the success of the recent surge in immune checkpoint blockades (ICB). Although the origin of neoantigens has been discussed extensively in published literature, the identification of neoantigens and their influence in clinical practice are largely ignored.

The application of neoantigens is rapidly becoming more widespread in clinical settings, not only just related to the development of tumor vaccine or adoptive cell therapy but also in the monitoring of clinical response in ICB and other therapies as well. The present Research Topic titled *“The Clinical Application of Neoantigens”* features 10 articles that reflect the clinical application of neoantigens and develop novel strategies for cancer therapy. This Research Topic includes mostly review articles about neoantigen-identified tools or methods, neoantigens as a basis for immunotherapies, and work using The Cancer Genome Atlas (TCGA), Gene Expression Omnibus (GEO) or own researchers collected datasets to identify novel biomarkers.

Hao et al. proposed a deep convolutional neural network named APPM (antigen presentation prediction model) to predict antigen presentation in the context of human leukocyte antigen (HLA) class I alleles. APPM is trained on large mass spectrometry (MS) HLA-peptides datasets and evaluated with an independent MS benchmark. Finally, they identified 16,000 putative neoantigens with the hallmarks of ‘drivers’. Generally, this study is only based on MS datasets, however, Next-Generation Sequencing (NGS) including RNA-sequencing (RNA-seq), whole-genome sequencing (WGS), and whole-exome sequencing (WES) is more frequently used for neoantigens. One has to additionally take into consideration already previously published work that proposed five major types of neoantigen with NGS, such alternative mRNA splicing (AS), chimeric RNAs (or fusion transcripts), circular RNAs (circRNAs), RNA editing, transposable elements (TEs), and human endogenous retroviruses (HERV) (1). Generally, numerous work about various bioinformatics pipelines and tools in this regard have been performed. Prof. Griffith, e.g., already outlined each step in the neoantigen workflow to predict high-quality immunogenic neoantigens (2).

Six review articles in the special issue focus on neoantigens as a basis for immunotherapies. Hereby, Dr. Zhong (Zhang et al.) summarized the latest advances in the classification of immunotherapy and the process of classification, identification, and synthesis of tumor-specific neoantigens, as well as their role in current cancer immunotherapy. Furthermore, prospects and existing problems of neoantigens are discussed, as is the long development cycle of neoantigen vaccine that is recognized as a primary obstacle to the application of vaccine, and the challenge of preparation and delivery of vaccines. Yu et al. introduced pyroptosis and ferroptosis as recently discovered types of programmed cell death (PCD) that are different from apoptosis, necrosis, and autophagy. They highlight that tumor cell neoantigens target tumor cells and cause pyroptosis or ferroptosis which might be an additional strategy for the future. Arnaud et al. provided an overview of the main strategies for T cell receptor (TCR)-engineering, described the selection and expansion of optimal carrier cells for TCR-adoptive T cell transfer (ACT) and discussed the next-generation methods for rapid identification of relevant TCR candidates for gene transfer therapy. Particularly CRISPR-Cas9 technology was recommended to verify TCR candidates for clinical practice. Zhu and Liu outlined the challenges of targeting neoantigens for cancer treatment. It is warranted to explore the combinatorial approaches with other immunotherapies, including checkpoint blockade therapies or conventional treatments, including chemoradiotherapies, kinase inhibitors, and anti-angiogenesis therapies. Gu et al. provided a clear picture of the clinical application of neoantigens in esophageal cancer (EC) from tumor-specific cancer vaccines and adoptive T cells to combination therapies. Liao and Zhang reported about neoantigen vaccines that were identified in preclinical and clinical trials and summarized the safety and efficacy of personalized cancer vaccines combined with ICBs in several cancer types. Most of the clinical trials are phase I or IB; only three phase II studies are recruiting.

Tumor antigens may be recognized by the immune system as non-self and elicit an immune response. Tumors with high TMB and/or MSI-H/dMMR may lead to an increase in neoantigens. Wang et al. therefore reviewed the potential application of tumor neoantigen burden (TNB) as a biomarker. The impact of high TNB and increased number of infiltrating immune cells on the efficacy of immunotherapies is discussed. Zou et al. summarized neoantigen load (NAL) which is similar to TNB, as a biomarker for predicting the anti-tumor effects of ICB. When NAL alone is insufficient to predict efficacy, its combination with other indicators can improve prediction efficiency. Liu et al. found that overexpression of *ERO1L* was associated with poor prognoses in patients with Lung Adenocarcinoma (LUAD). Overexpression of *ERO1L* was indicative of a hypoxia-induced immune-suppressive TIME, which was shown to confer resistance to immunotherapy in patients with LUAD.

In summary, this Research Topic highlights that tumor neoantigens play an essential role in antitumor immunity and successful cancer immunotherapies regardless of cancer vaccine alone or combinations with ICB. With the development of bioinformatics pipelines and tools, more novel neoantigens including circRNA, AS, fusion, and TE, are identified. Thereby, neoantigen-based tailored therapies can be widely performed in various cancers soon. Neoantigens could serve as potential biomarkers for cancer patients treated with immunotherapies. In the next decades, we still need to investigate more and more novel neoantigens as potential prognostic, predictive, and safety biomarkers for cancer patients treated with immunotherapies alone and/or conventional therapies such as RT and CT (**Figure 1**). Let’s go ahead with translational research of neoantigens for cancer!

**Figure 1:**
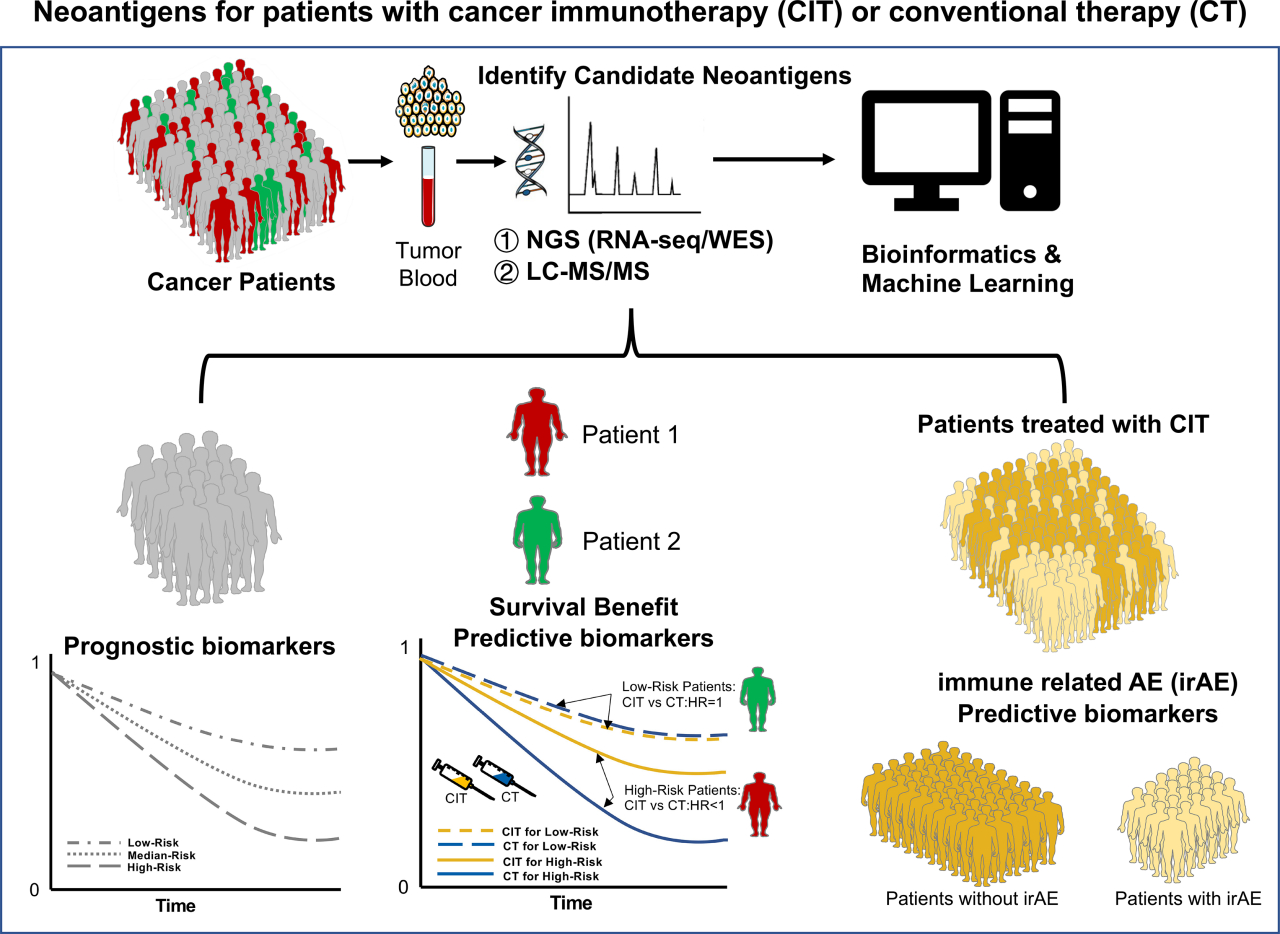
Neoantigens as potential biomarkers for cancer patients treated with immunotherapies and/or conventional therapies. Neoantigens are mostly derived from mutations in tumor cells. The candidate neoantigen can be identified from tumor tissue and blood samples with bioinformatics analysis ①. Next-Generation Sequencing (NGS), including RNA-sequencing (RNA-seq), whole-genome sequencing (WGS), and whole-exome sequencing (WES); ②. Liquid chromatography-mass spectrometry (LC-MS). They can serve as three major potential biomarkers. First, as a prognostic biomarker which indicates an increased (or decreased) likelihood of a future clinical event, disease recurrence, or progression in an identified population. Predictive biomarkers are used to identify individuals who are more likely than similar individuals without the biomarker to experience a favorable or unfavorable effect from exposure to a medical product or an environmental agent. Safety biomarkers are measured before or after an exposure to a medical product or an environmental agent to indicate the likelihood, presence, or extent of toxicity as an adverse effect. Figure modified with text, markings (stars), and annotation after adapted from Servier Medical Art by Servier, licensed under a Creative Commons Attribution 3.0 Unported License. Original photo adapted from https://smart.servier.com/smart_image.

## Authors Contributions

All authors listed have made a substantial, direct, and intellectual contribution to the work, and approved it for publication.

## Funding

This work is supported by the National Natural Science Foundation of China (NSFC) (Grant No. 82060475), Research Programs of Science and Technology Commission Foundation of Guizhou Province (Grant Nos. QKHJC[2020]1Z062), Research Programs of Science and Technology Commission Foundation of Zunyi City (Grant Nos. HZ2019-11 and HZ2019-07), Research Programs of Health Commission Foundation of Guizhou Province (Grant Nos. gzwjkj2019-1-073 and gzwjkj2019-1-172), Lian Yun Gang Shi Hui Lan Public Foundation (Grant No. HL-HS2020-92). We acknowledge Servier Medical Art (https://smart.servier.com) for providing images of cell, human, and cartoon components.

## Conflict of Interest

The authors declare that the research was conducted in the absence of any commercial or financial relationships that could be construed as a potential conflict of interest.

## Publisher’s Note

All claims expressed in this article are solely those of the authors and do not necessarily represent those of their affiliated organizations, or those of the publisher, the editors and the reviewers. Any product that may be evaluated in this article, or claim that may be made by its manufacturer, is not guaranteed or endorsed by the publisher.
